# Subinguinal microsurgical varicocelectomy vs. percutaneous embolization in infertile men: Prospective comparison of reproductive and functional outcomes

**DOI:** 10.1186/s12610-017-0055-x

**Published:** 2017-06-08

**Authors:** Elie Bou Nasr, Mouath Binhazzaa, Thierry Almont, Pascal Rischmann, Michel Soulie, Eric Huyghe

**Affiliations:** Department of Urology, Toulouse university hospital, 1, avenue Jean Poulhès – TSA 50032, 31059 Toulouse cedex 9, France

**Keywords:** Varicocele, Semen analysis, Subinguinal varicocelectomy, Percutaneous embolization, Varicocèle, Spermogramme, Varicocèlectomie sous-inguinale, Embolisation Percutanée

## Abstract

**Background:**

Varicocele is a condition characterized by dilated, tortuous veins within the pampiniform venous plexus of the scrotal sac. Presence of varicocele is associated with an increased risk of alteration of semen parameters. The objective of this study was to compare the current standard in varicocele treatment procedures: sub-inguinal microscopic ligation to percutaneous embolization in terms of semen parameters improvement, fertility, and morbidity at the university hospital of Toulouse (France). Seventy six patients with clinical varicocele, alteration of semen parameters and infertility, underwent either procedure (microsurgery in 49 case performed by a single surgeon and embolization in 27 cases) and were prospectively analyzed. Outcome measures were: semen parameters, spontaneous pregnancies, pain, side effects, recovery time and overall satisfaction. All patients were contacted in January 2015 in order to determine reproductive events.

**Results:**

Preoperatively, there was no difference in clinical and biological items between the two groups. Postoperatively, on the overall population, there was a significant improvement of sperm concentration at 3, 6, 9 and 12 months (*p* = <0.001, <0.001, 0.012, 0.018) and sperm motility at 6 months (*p* = 0.002). The sperm concentration was higher at 6 months in the percutaneous embolization group (13.42, vs. 8.1×10^6^/ml; *p* = 0.043). With a median follow-up of 4 years, 27 pregnancies occurred (spontaneous pregnancy rate of 35.5%).

There was no significant difference between procedures on the sperm quality, pregnancy rate, and the overall satisfaction. Patients undergoing percutaneous embolization reported a faster recovery time (*p* = 0.002) and less postoperative pain (*p* = 0.007).

**Conclusion:**

Our study shows that percutaneous embolization seems to be an equivalent alternative to sub-inguinal microscopic ligation in term of sperm quality improvement, pregnancy rate, and overall satisfaction with a slight advantage on post-operative morbidity.

## Résumé

**Contexte:**

La varicocèle est une affection caractérisée par une dilatation et tortuosité des veines du plexus veineux pampiniforme du sac scrotal. La présence de varicocèle est associée à un risque accru d'altération des paramètres du sperme. Cette étude vise à comparer la norme actuelle des procédures de traitement par varicocèle: la ligature microscopique sous-inguinale à l'embolisation percutanée en termes d'amélioration des paramètres spermatiques, de fertilité et de morbidité dans l'hôpital universitaire de Toulouse(France). Soixante-seize patients atteints de varicocèle clinique, d'altération des paramètres spermatiques et d'infertilité ont subi une intervention (microchirurgie dans 49 cas réalisées par un seul urologue et embolisation dans 27 cas) et ont fait l'objet d'une analyse prospective. Les données étudiées sont les suivantes: paramètres du sperme, grossesses spontanées, douleur, effets secondaires, temps de récupération et satisfaction globale. Tous les patients ont été contactés en janvier 2015 afin de déterminer les grossesses spontanées.

**Résultats:**

En préopératoire, il n’existait aucune différence sur les items cliniques et biologiques entre les deux groupes. Sur la population globale, on observe une amélioration significative de la concentration spermatique à 3, 6, 9 et 12 mois (*p* = <0,001, <0,001, 0,012, 0,018) et de la motilité à 6 mois (*p* = 0,002). La concentration de spermatozoïdes était plus élevée à 6 mois dans le groupe d'embolisation percutané (13,42 vs 8,09; *p* = 0,043). Avec un suivi médian de 4 ans, 27 grossesses sont survenues (taux de grossesse spontanée de 35.5%).

Il n'y avait pas de différence significative entre les procédures sur la qualité du sperme, le taux de grossesse et la satisfaction globale. Les patients traités par embolisation percutanée ont rapporté un temps de récupération plus rapide (*p* = 0,002) et moins de douleur postopératoire (*p* = 0,007).

**Conclusion:**

Notre étude montre que l'embolisation percutanée semble être une alternative équivalente à la ligature microscopique sous-inguinale en terme d'amélioration de la qualité du sperme, du taux de grossesse et de la satisfaction globale avec un léger avantage sur la morbidité post-opératoire.

## Background

Varicocele is a condition characterized by dilated, tortuous veins within the pampiniform venous plexus of the scrotal sac. It is a fairly common condition, accounting for 35% of men with primary infertility and 75–81% of men with secondary infertility [1]. It is clearly established that presence of varicocele is associated with an increased risk of alteration of sperm parameters, although the mechanisms have not yet been fully described , it is likely to be multifactorial. Recent studies showed that it resulted mainly in higher levels of reactive oxygen species (ROS) [2–4]. This excessive ROS is associated with sperm DNA fragmentation, which may mediate the clinical manifestation of poor sperm function and fertilization outcome related to varicocele [5, 6].

For Shiraishi K. et al. the oxidative stress is due to the increased scrotal temperature and not directly associated to varicocele grade, causing a disturbance of the oxidative stress scavenging system in infertile men with varicoceles [7, 8].

Two meta-analyses showed that semen improvement is usually observed after surgical correction [9, 10] and that varicocelectomy can reverse sperm DNA damage [11].

The sub-inguinal microsurgery (SIS) is the treatment of reference as it shows lower rates of relapse, and does not expose to vital complications [12]. In recent years, a new radiological technique, called percutaneous embolization (PE) was put forward as a non-surgical alternative. However, so far, there is a paucity of comparative studies the literature between these two techniques [13, 14]. Therefore, the question of which procedure (PE or SIS) is more effective remains debated. Moreover, surprisingly for these mini-invasive procedures, no comparative study analyzed postoperative morbidity, pain and recovery.

Considering this ongoing controversy, we aimed at comparing prospectively the both procedures regarding reproductive outcomes as well as functional results.

## Methods

We prospectively analyzed preoperatively and post-operatively (at 1, 3, 6, 9 and 12 months) patients with clinical varicocele, alteration of semen parameters and infertility, undergoing either procedure (Microsurgery in 49 cases and Embolization in 27 cases) Outcome measures were: semen parameters, pregnancies, pain, side effects, recovery time and overall satisfaction. Subsequently, all patients were contacted by telephone in January 2015 (with a median delay of 4 years after the procedure) in order to determine the reproductive events.

A prospective series of patients treated for varicocele at the University Hospital of Toulouse, between January 2007 and December 2013 was assessed. Inclusion criteria were: 1) clinical varicocele; 2) couple infertility for at least one year, 3) at least one abnormal semen parameter confirmed by two semen analyses within an interval of three weeks, 4) absence of other contributing male risk factors and 5) female partner considered fertile at the date of evaluation. Azoospermic patients were excluded from this study.

The decision to treat was taken by an andrology committee, including urologists, andrologists, gynecologists and biologists. The choice of the technique was left to the patient after clear and comprehensive information on the advantages and complications of each. It is important to note that the two procedures being equally covered by the French healthcare system, financial issues did not play a part in patient’s decision.

All Patients underwent a pre-operative assessment including clinical history, physical examination, hormonal screening and a scrotal doppler ultrasonography (DUS). Varicocele was classified as: Subclinical if detected only by DUS, Grade 1 if the varicocele was palpable during Valsalva maneuver, Grade 2 if it was palpable at rest and Grade 3 if it was visible and palpable at rest, according to the Dubin grading system [15]. Parameters noted at DUS were the size of varicocele, and the length of reflux following Valsalva maneuver (cutoff at 2 s).

Patients had at least two preoperative semen analyses, performed according to the World Health Organization references guidelines for semen analyses [16]. It is important to note that as the study ran between January 2007 and December 2013, two WHO values for semen analysis were used.

Normality of karyotype and/or microdeletion of the Y chromosome were verified when indicated (karyotype if count <5 million/ml and microdeletion in chromosome y if < 1 million/ml on at least 2 exams). Finally, among the 76 included patients, 49 (64.5%) underwent SIS (Group 1) and 27 (35.5%) PE (Group 2). All SIS were performed by the same specialized urologist. Embolizations were realized by two interventional radiologists using the same technique.

All patients were assessed at 1 month postoperatively. Semen analyses were performed at 3, 6, 9 and 12 months. The parameters analyzed were: total sperm count, progressive motility 1 h following ejaculation, vitality, normal forms: according to the classification of David [17], and total motile sperm count (TMSC).

Finally, we approached the patients on January 2015 to reply to a phone questionnaire to evaluate: a) patient-physician relationship, b) postoperative complications, c) treatment impact on daily life following the procedure, d) duration of work absence, e) postoperative pain. The questionnaire was reviewed and approved by the Toulouse committee for person protection in biomedical research.

The patients being followed as couples in our fertility center, the data concerning spontaneous pregnancy was retrieved from patient’s medical file. Pain was assessed using an analogic scale (1 meaning a mild postoperative pain and 5 a severe pain).

### Statistical analysis

Descriptive statistics were performed for semen health parameters at the 3, 6, 9 and 12 month stages. A Chi-square test was used to examine the association between techniques (SIS and PE), side of the varicocele, type of infertility and grade of varicocele. A paired T-test or Wilcoxon test was performed to assess the evolution of sperm quality between the pre-operational level and the 3, 6, 9 and 12 month review. A Student T-test or Mann-Whitney test was used to compare the outcomes between both treatments at each moment (0, 3, 6, 9 and 12 months). ANOVA was performed on postoperative semen parameters (with logarithmic transformation when needed) to assess global difference between both treatments.

All tests were 2-sided, and no adjustment for multiple comparisons was made. *P-values* < 0.05 were considered statistically significant. Analyses were performed using Stata software (version 12; Stata Corp., College Station, Tex).

## Results

Preoperatively, there was no significant clinical or biological difference between both groups. Primary infertility was the most common type of infertility, in89.8% of Group 1 (44 patients) and 88.8% of Group 2 (24 patients). The varicocele was limited to the left side in the majority 83.6% of Group 1 (41 patients) and 100% of Group 2; (27 patients), only 8 patients (16.3%) were treated bilaterally, by SIS in all the cases. In group 2, no patient had right side varicocele. The varicocele grades were as follows: 12% grade 1(9 patients), 26% grade 2 (20 patients) and 62% grade 3(47 patients).

On the whole series, compared to the preoperative semen analysis, there was a significant improvement of sperm count at 3, 6, 9 and 12 months postoperatively (*p* = <0.001, <0.001, 0.012, 0.018 respectively) and a significant increase in sperm progressive motility only at 6 months (*p* = 0.002). The TMSC was significantly improved at 3, 6, 9 months (*p* = <0.001, <0.001, 0.012 respectively). No significant change in vitality or in the percentage of normal forms was noted (Table 1).

**Table 1:** Evolution of semen parameters on the overall population

Description	Pre-op^a^	3 months	6 months	9 months	12 months	Last exam^b^
Abstinence delay(day)(n) (p, Wilcoxon)	3.8 ± 0.8 (75)	3.8 ± 1.0(68) 0.246	3.7 ± 0.9(48) 0.543	3.7 ± 0.7(27) 0.115	4.0 ± 2.4(29) 0.815	3.7 ± 1.7(73) 0.221
Semen volume (ml) (p, Wilcoxon)	4.3 ± 1.8 (75)	4.0 ± 1.7(68) 0.670	4.5 ± 2(48) 0.085	4.1 ± 2(27) 0.775	3.8 ± 1.9(29) 0.494	4.5 ± 2.0(73) 0.457
Sperm count x10^6^/ml(n) (p, Wilcoxon)	7.1 ± 10(75)	12.3 ± 13.6(68) <0.001	10.2 ± 9.8(48) <0.001	12.6 ± 14(27) 0.003	11.2 ± 12.6(29) 0.018	12.4 ± 13.2(73) <0.001
Motility%(n) (p, t-test)	22.7 ± 11.2(70)	24.9 ± 13.6(67) 0.186	27.4 ± 13.5(48) 0.002	22.4 ± 12.5(27) 0.215	20.8 ± 13.2(28) 0.631	25.7 ± 14.8(72) 0.048
Vitality%(n) (p, Wilcoxon)	60.3 ± 14.7(70)	57.8 ± 17(65) 0.906	57.9 ± 17.7(48) 0.472	51.6 ± 17.4(27) 0.558	55.1 ± 20.3(28) 0.904	58 ± 18.9(71) 0.490
Normal forms^c^%(n) (p, Wilcoxon)	5.7 ± 9.4(39)	7.4 ± 7.6(35) 0.072	4.4 ± 4.1(30) 0.776	4.9 ± 5.3(15) 0.777	10.8 ± 8(12) 0.176	8.5 ± 12.2(40) 0.840
TMSCx10^6^(n) (p, Wilcoxon)	6.7 ± 9.9(73)	14.3 ± 18.74(67) <0.001	13.8 ± 15.9(48) <0.001	12.3 ± 17.6(27) 0.004	10.1 ± 15.1(28) 0.248	10.2 ± 13.9(39) 0.100

^a^Pre operative values

^b^Represents the mean of the last semen analysis available for each patient

^c^The Classification of David was used to evaluate normal forms

The mean of each value is reported +/- SD

Reported data are rounded up

There was a significant higher sperm concentration at 6 months postoperatively in group 2 (13.42 ×10^6^/ml, vs. 8.09 ×10^6^/ml; *p* = 0.043) using the Mann Whitney test) (Table 2). This difference still persisted in ANOVA on logarithmic transformation of sperm concentration at 6 months (*p* = 0.01), and on the 3-9 months period (*p* = 0.040). There was however no significant difference in other post-operative sperm parameters between the two groups (Tables 3 and 4)

**Table 2:** Sperm count and motility: surgery vs. embolization

Sperm countx10^6^/ml	Surgery	Embolization	P-value Mann-Whitney	Motility%	Surgery	Embolization	*P*-value t-test
Pre-op (n)	6.2 ± 10.7 (48)	8.6 ± 8.6 (27)	0.070	Pre-op (n)	22.4 ± 12.7(45)	23.3 ± 7.8 (25)	0.748
3 months (n)	10.3 ± 12.5 (43)	15.6 ± 14.9 (25)	0.112	3 months (n)	25.9 ± 13.7(42)	23.3 ± 13.6 (25)	0.457
6 months (n)	8.1 ± 9.3 (29)	13.4 ± 9.9 (19)	0.043	6 months (n)	27.2 ± 14.4(29)	27.6 ± 12.4 (19)	0.916
9 months (n)	12.0 ± 15.8 (13)	13.1 ± 12.7 (14)	0.544	9 months (n)	24.2 ± 10.8 (13)	20.6 ± 14.2 (14)	0.468
12 months (n)	9.4 ± 12.1 (18)	14.0 ± 13.4 (11)	0.126	12 months (n)	21.7 ± 13.9 (17)	19.5 ± 12.6 (11)	0.676
Last exam^a^(n)	11.2 ± 12.8 (46)	14.3 ± 13.7 (27)	0.183	Last exam(n)	26.6 ± 15.3 (45)	24.4 ± 14.0 (27)	0.536

The mean of each value is reported +/- SD

Reported data are rounded up

^a^Represents the mean of the last semen analysis available for each patient

**Table 3:** Vitality and normal forms: surgery vs. embolization

Vitality%	Surgery	Embolization	*P*-value Mann-Whitney	Normal Forms^b^%	Surgery	Embolization	*P*-value Mann-Whitney
Pre-op (n)	62.3 ± 14.0 (45)	56.7 ± 15.6 (25)	0.063	Pre-op (n)	6.4 ± 10.9 (25)	4.3 ± 5.9 (14)	0.488
3 months (n)	60.7 ± 14.2 (40)	53.2 ± 20.9 (25)	0.131	3 months (n)	6.1 ± 6.4 (22)	9.6 ± 9.2 (13)	0.244
6 months (n)	59.9 ± 14.6 (29)	54.8 ± 21.7 (19)	0.548	6 months (n)	4.1 ± 4.3 (20)	4.9 ± 3.7 (10)	0.413
9 months (n)	54.5 ± 12.5 (13)	49.0 ± 21.0 (14)	0.698	9 months (n)	6.3 ± 2.9 (7)	3.8 ± 6.8 (8)	0.041
12 months (n)	54.3 ± 22.9 (17)	56.4 ± 16.6 (11)	0.724	12 months (n)	10.9 ± 9.6 (8)	10.8 ± 4 (4)	0.733
Last exam^a^ (n)	58.1 ± 18.6 (44)	57.8 ± 19.7(27)	0.844	Last exam(n)	6.7 ± 7.1(27)	12.2 ± 18.7 (13)	0.393

The mean of each value is reported +/- SD

Reported data are rounded up

^a^Represents the mean of the last semen analysis available for each patient

^b^The Classification of David was used to evaluate normal forms

**Table 4:** TMSC: surgery vs. embolization

Total motile sperm count x10^6^(TMSC)	Surgery	Embolization	*P*-value Mann-Whitney
Pre-op (n)	5.7 ± 10.3 (48)	8.5 ± 9.2 (25)	0.263
3 months (n)	11.9 ± 15.8 (42)	18.4 ± 22.6 (25)	0.171
6 months (n)	10.5 ± 13.2 (29)	18.7 ± 18.7 (19)	0.079
9 months (n)	13.4 ± 22.2 (13)	11.2 ± 12.6 (14)	0.760
12 months (n)	8.1 ± 10.5 (17)	13.1 ± 20.5 (11)	0.401
Last exam^a^ (n)	9.1 ± 11.1 (22)	11.5 ± 17.1 (17)	0.745

The mean of each value is reported +/- SD

Reported data are rounded up

^a^Represents the mean of the last semen analysis available for each patient

All patients responded to the telephone contact survey. The majority of individuals in both groups were satisfied about the amount and content of information given preoperatively (82% in Group 1, vs.74% in Group 2; *p* = 0.778).

Three patients in Group 1 had a post-operative complication (skin infections in 2 cases and scrotal hematoma in 1 case) and none in Group 2 (*p* = 0.549). Patients undergoing percutaneous embolization reported a faster recovery time with a total number of patients requesting a sick leave significantly lower in Group 2 (57% in Group 1 vs. 25% in Group 2; *p* = 0.002); and neither group reported any negative impact on sports activity The percentage of patients in Group 1 with a pain level above 2/5 was significantly higher than in Group 2 (44% in Group 1, vs. 22% in Group 2; *p* = 0.007), and there were no patients with pain level above2/5 in Group 2. Concerning spontaneous pregnancy, both groups had similar pregnancy rates (36.7% Group 1 vs. 33.3% Group 2; p-value = 0.778), the event occurring after a mean delay of 6.4 months. The Overall satisfaction about the treatment and post-operative care was not different in both groups (80% Group 1, vs. 77% in Group 2; *p* = 0.877) (Table 5).

**Table 5:** postoperative comparison surgery vs. embolization

	Surgery	Embolization	*P*-value
Complications (n)	3^a^	0	0.549
Pain (mean/5)	2.1	1.5	0.034
Sick leave (mean days)	6.8	1.5	0.002
Satisfaction (mean/5)	4.5	4.6	0.877
Pregnancy (n)	18(36.7%)	9(33.3%)	0.778

Reported data are rounded up

^a^Wound infection in 2 cases and scrotal hematoma in 1 case

## Discussion

In 2006, Evers and Collins published a famous meta-analysis in the Cochrane database, indicating the discredit on varicocele repair [18]. This meta-analysis included 7 randomized control trials. The judgment criterion was the birth rate. Based on a non-significant difference in birth rate between the two groups (22% in the treatment group, vs.19% in the control group), the authors concluded that “There is no evidence that treatment of varicocele in men from couples with otherwise unexplained subfertility improves the couple's chance of conception”. Following this publication, several societies, including the European Association of Urology (EAU) revised their guidelines, considering: Treatment of varicocele to achieve pregnancy in infertile couples remains controversial and all studies to date have been subject to criticism [19].

However, critical analysis of the included studies pointing out several biases, notably that infraclinical varicoceles and patients with normal semen parameters were included. Since that time, several meta-analyses, excluding the biased studies [20] or including complementary data from good quality observational studies [10] have been published, showing a significant improvement in the pregnancy rate.

Finally, a new randomized controlled trial including 148 infertile men with a clinical varicocele, and altered semen parameters was published by Abdel-meguid et al. [21] They observed a significant difference in favor of varicocele treatment (OR =3.04 (1.33-6.95)). Subsequently, Baazzem et al. published a new meta-analysis that found an OR at 2.69 (1.16 – 6.24) between varicocele treatment and surveillance [9]. They also clearly highlighted the impact of varicocele treatment on all semen parameters (compared to controls, OR = 12.3 (9.45 – 15.19) for sperm concentration, 0.86 (7.07 – 14.65) for total motility, 9.69 (4.86 – 14.52) for progressive motility [5]. This positive impact of varicocele treatment on semen parameters has been supported by two recent meta-analyses [22, 23]. Moreover, Baazzem et al. included in their study new interesting end points that were not analyzed previously, and that were improved following the treatment (oxidative stress, sperm DNA damage, sperm ultrastructure) [5]. In conclusion, the benefits of varicocele treatment seem much more evident than in the last decade. Considering the quality of studies rendered so far, it seemed difficult to come up with a definite conclusion, the last meta-analysis from the Cochrane (2012) finally favored treatment, with a combined OR 2.39 (95% CI 1.56 to 3.66), and a calculated number of patients needing to be treated for an additional beneficial outcome of 7 [24].

Concerning the techniques of varicocele treatment, recent comparisons figure that sub-inguinal microsurgical technique is the treatment of choice, allowing the lowest recurrence rate (0.8 to 4%), without exposing to severe complications, [12, 22] recommending it also as first line treatment [25]. Shiraishi et al. compared the results and complications of retroperitoneal, microsurgical subinguinal, and high inguinal approaches in the treatment of varicoceles [26], and concluded that significant postoperative improvements in sperm concentration and motility were observed after all approaches, but results were observed sooner and showed higher sperm concentration and motility after the use of the microsurgical approaches.

However, data concerning the percutaneous embolization should be regarded cautiously, as they remain scarce. A recent radiological study, without surgical group concluded arbitrarily that “the only situation in which PE has failed to show itself equal or superior to other established techniques is in the case of bilateral varicocele” placing PE as a new gold standard in most cases [27].

Considering this ongoing debate, our study aimed to compare thoroughly the results of SIS and PE, in order to draw conclusions about how to treat varicocele optimally. In this study, both groups showed a significant improvement in sperm count, sperm progressive motility (only at 6 months) and the TMSC (at 3, 6, 9 months) postoperatively. There was no significant difference according to postoperative semen parameters between the two groups, except a higher sperm concentration at 6 months postoperatively in group 2. However, it must be noted that preoperatively, there was a tendency for a higher sperm concentration in group 2 (8.63 ×10^6^/ml, vs. 6.23 ×10^6^/ml in group 1). In addition, the postoperative TMSC which is a parameter well correlated with fecundability was statistically non-significant between the both groups at any times. Therefore, our results fully agree with many studies that both the procedures provide similar increase in sperm quality [28]. Also, studies favoring higher results after SIS than PE only show marginal differences [14].

Concerning the pregnancy rate, we observed roughly the same result in both groups. Analysis of the literature shows that one third of couples achieve a spontaneous pregnancy which is frequently reported by studies that had sufficient follow-up [29]. Nevertheless, contrarily to the generally admitted idea that following varicocele treatment, semen improvement occurs progressively during the first year, we observed for all parameters (concentration, motility, vitality, normal forms and TMSC) a sharp rebound at 3 months, followed by a slight decrease but a level at the last analysis remaining higher than preoperatively. Recently Al Bakri *et al* reported similarly, that sperm parameters improve by 3 months following varicocele repair and then no more improvement by 6 months or longer [30]. The cause of such phenomenon remains unclear.

Concerning post-operative complications, it is difficult to know from the literature the frequency of complications for each procedure, due to heterogeneous report of them (no use of validated classification, difficulty to identify mild side effects, inclusion of recurrence). With only 3 patients reporting mild post-operative complications in group 1 and no one in group 2, we confirm that the two techniques are safe procedures. Our methodology does not enable us to verify if the PE exposes to higher recurrence rates than SIS [31]. On the other hand, we did not observe that hydrocele is more frequent after subinguinal technique as it is reported in the literature [32, 33]. Obviously, the number was too low to draw any meaningful conclusions but the key message is that complications are minor and of little consequence with these 2 procedures.

Post-operative pain and its duration seemed less in the PE group, with no patient reporting anything above 2/5 in this group. Many patients in the SIS group describe uncomfortable feeling at the incision site, rather than pain, especially at the end of the day without any evidence of abnormal clinical or radiological examination. Obviously, we found that a pain level above 2 was associated with longer sick leave in the SIS group. Taking this into account, one may argue that patients who care about an expedient recovery may be advised to perform PE rather than SIS; however, it is difficult to recommend it as the consumption of analgesia was not assessed.

Overall satisfaction was high in both groups concerning the pre-operative information as well as post-operative outcomes. This high satisfaction may be understood, for the preoperative assessment, by the systematic discussion with the couple of the two modalities of treatment, together with the use of an informational booklet written by the French Urology Association, and the fact that the patient participated in the choice of treatment.

For the postoperative assessment, satisfaction may reflect the occurrence of a pregnancy without need of assisted reproductive techniques (ART) for one in 3 patients, and in the absence of pregnancy, the mini-invasiveness of treatments, together with an improvement of semen quality that may have a positive impact on the outcome of ART [34].

There were however a few limitations that must be noted when interpreting our results, mainly the small sample size, the fact that these data come from only one center, the absence of randomization of treatment and subjectivity of phone interview questionnaire. Qualitative results must always be interpreted with caution. Pain endurance, pain intensity, what one individual considers a hindrance to activity and return to normal life are all eminently subjective variables.

It is also important to note that 8 patients (16.3%) underwent bilateral SIS while all PE were left sided. This difference may affect the comparability and interpretation of our results.

Another difficulty comes from patient’s compliance to perform a semen collection at all the designated times. One in three patients only achieved the one-year full protocol, and we had difficulties to collect the semen analysis at 9 and 12 month. To deal with this issue, we created the variable “last analysis”, meaning the last semen analysis performed for each patient, but obviously, interpretation of results should again be viewed with caution as it renders difficult to analyze precisely fluctuations within the study period.

Our study does not compare the two techniques from a medico-economic point of view. Many studies have shown that in various medical systems, varicocele surgery is more cost effective than assisted reproduction techniques [31, 35]. However, we found that it was not valuable to compare the two techniques, taking into account that costs are greatly dependent from one country to another. It must be noted, however, that the 2 techniques are always performed in outpatient day-care, and thus, there is no obvious difference in hospitalization cost between the PE and SIS [33].

## Conclusion

The treatment of clinical varicocele for infertile men, either by sub-inguinal microsurgery approach or percutaneous embolization shows globally equivalent outcomes, notably in terms of sperm quality, pregnancy rate and satisfaction. However, after PE recovery time is faster and pain is less than sub-inguinal microsurgery. These results confirm that PE is a safe and efficient alternative to SIS. Considering the various limitations of our study, we cannot go further into recommendations, and so far, both options can be chosen freely according to surgeon experience and patient preferences. Further studies are strongly recommended in the field with larger sample size to ascertain conclusions drawn from this study.

## Abbreviations

ANOVA: analysis of variance
ART: assisted reproductive techniques
DUS: doppler ultrasonography
PE: percutaneous embolization
ROS: reactive oxygen species
SIS: sub-inguinal microsurgery
TMSC: total motile sperm count
WHO: World health organization

## References

[1] Gorelick JI, Goldstein M (1993). Loss of fertility in men with varicocele. Fertil Steril.

[2] Kass EJ, Reitelman C (1995). Adolescent varicocele. Urol Clin North Am.

[3] Practice Committee of the American Society for Reproductive M, Society for Male R, Urology (2014). Report on varicocele and infertility: a committee opinion. Fertil Steril.

[4] Mostafa T, Anis TH, El-Nashar A, Imam H, Othman IA (2001). Varicocelectomy reduces reactive oxygen species levels and increases antioxidant activity of seminal plasma from infertile men with varicocele. Int J Androl.

[5] Zini A, Azhar R, Baazeem A, Gabriel MS (2011). Effect of microsurgical varicocelectomy on human sperm chromatin and DNA integrity: a prospective trial. Int J Androl.

[6] Cho CL, Esteves SC, Agarwal A (2016). Novel insights into the pathophysiology of varicocele and its association with reactive oxygen species and sperm DNA fragmentation. Asian J Androl.

[7] Shiraishi K, Takihara H, Naito K (2009). Testicular volume, scrotal temperature, and oxidative stress in fertile men with left varicocele. Fertil Steril.

[8] Shiraishi K, Takihara H, Matsuyama H (2010). Elevated scrotal temperature, but not varicocele grade, reflects testicular oxidative stress-mediated apoptosis. World J Urol.

[9] Baazeem A, Belzile E, Ciampi A, Dohle G, Jarvi K, Salonia A (2011). Varicocele and male factor infertility treatment: a new meta-analysis and review of the role of varicocele repair. Eur Urol.

[10] Agarwal A, Deepinder F, Cocuzza M, Agarwal R, Short RA, Sabanegh E (2007). Efficacy of varicocelectomy in improving semen parameters: new meta-analytical approach. Urology.

[11] Zini A, Dohle G (2011). Are varicoceles associated with increased deoxyribonucleic acid fragmentation?. Fertil Steril.

[12] Schiff J, Kelly C, Goldstein M, Schlegel P, Schelgel P, Poppas D (2005). Managing varicoceles in children: results with microsurgical varicocelectomy. BJU Int.

[13] Gómez Beltrán O, Garrido Pérez JI, García Ceballos A, Escassi Gil A, Vargas Cruz V, Lasso Betancor CE (2013). Open surgery, laparoscopic Palomo varicocelectomy and embolization in children with varicocele. Cir Pediatr.

[14] Yavetz H, Levy R, Papo J, Yogev L, Paz G, Jaffa AJ (1992). Efficacy of varicocele embolization versus ligation of the left internal spermatic vein for improvement of sperm quality. Int J Androl.

[15] Dubin L, Amelar RD (1977). Varicocelectomy: 986 cases in a twelve-year study. Urology.

[16] Cooper TG, Noonan E, von Eckardstein S, Auger J, Baker HWG, Behre HM (2010). World Health Organization reference values for human semen characteristics. Hum Reprod Update.

[17] Auger J, Eustache F (2000). Standardisation de la classification morphologique des spermatozoïdes humains selon la méthode de David modifiée. Andrologie.

[18] Evers JL, Collins JA (2004). Surgery or embolisation for varicocele in subfertile men. Cochrane Database Syst Rev.

[19] Dohle GR, Colpi GM, Hargreave TB, Papp GK, Jungwirth A, Weidner W, Infertility EAUWGoM (2005). EAU guidelines on male infertility. Eur Urol.

[20] Ficarra V, Cerruto MA, Liguori G, Mazzoni G, Minucci S, Tracia A (2006). Treatment of varicocele in subfertile men: The Cochrane Review--a contrary opinion. Eur Urol.

[21] Abdel-Meguid TA, Al-Sayyad A, Tayib A, Farsi HM (2011). Does varicocele repair improve male infertility? An evidence-based perspective from a randomized, controlled trial. Eur Urol.

[22] Wang J, Xia S-J, Liu Z-H, Tao L, Ge J-F, Xu C-M (2015). Inguinal and subinguinal micro-varicocelectomy, the optimal surgical management of varicocele: a meta-analysis. Asian J Androl.

[23] Nork JJ, Berger JH, Crain DS, Christman MS (2014). Youth varicocele and varicocele treatment: a meta-analysis of semen outcomes. Fertil Steril.

[24] Kroese ACJ, de Lange NM, Collins J, Evers JLH (2012). Surgery or embolization for varicoceles in subfertile men. Cochrane Database Syst Rev.

[25] Kim KH, Lee JY, Kang DH, Lee H, Seo JT, Cho KS (2013). Impact of surgical varicocele repair on pregnancy rate in subfertile men with clinical varicocele and impaired semen quality: a meta-analysis of randomized clinical trials. Korean J Urol.

[26] Shiraishi K, Oka S, Ito H, Matsuyama H (2012). Comparison of the results and complications of retroperitoneal, microsurgical subinguinal, and high inguinal approaches in the treatment of varicoceles. J Androl.

[27] Cassidy D, Jarvi K, Grober E, Lo K (2013). Varicocele surgery or embolization: Which is better?. Can Urol Assoc J.

[28] Dewire DM, Thomas AJ, Falk RM, Geisinger MA, Lammert GK (1994). Clinical outcome and cost comparison of percutaneous embolization and surgical ligation of varicocele. J Androl.

[29] Jungwirth A, Giwercman A, Tournaye H, Diemer T, Kopa Z, Dohle G, et al. European Association of Urology Working Group on Male I. European Association of Urology guidelines on Male Infertility: the 2012 update. Eur Urol. 2012;62(2):324-32. 10.1016/j.eururo.2012.04.048 22591628

[30] Al Bakri A, Lo K, Grober E, Cassidy D, Cardoso JP, Jarvi K (2012). Time for improvement in semen parameters after varicocelectomy. J Urol.

[31] Diegidio P, Jhaveri JK, Ghannam S, Pinkhasov R, Shabsigh R, Fisch H (2011). Review of current varicocelectomy techniques and their outcomes. BJU Int.

[32] Cayan S, Shavakhabov S, Kadioğlu A (2009). Treatment of palpable varicocele in infertile men: a meta-analysis to define the best technique. J Androl.

[33] Bechara CF, Weakley SM, Kougias P, Athamneh H, Duffy P, Khera M (2009). Percutaneous treatment of varicocele with microcoil embolization: comparison of treatment outcome with laparoscopic varicocelectomy. Vascular.

[34] Haydardedeoglu B, Turunc T, Kilicdag EB, Gul U, Bagis T (2010). The effect of prior varicocelectomy in patients with nonobstructive azoospermia on intracytoplasmic sperm injection outcomes: a retrospective pilot study. Urology.

[35] Meng MV, Greene KL, Turek PJ (2005). Surgery or assisted reproduction? A decision analysis of treatment costs in male infertility. J Urol.

